# Biocompatible Probes Based on Rare-Earth Doped Strontium Aluminates with Long-Lasting Phosphorescent Properties for In Vitro Optical IMAGING

**DOI:** 10.3390/ijms23063410

**Published:** 2022-03-21

**Authors:** David G. Calatayud, Teresa Jardiel, Erica Cordero-Oyonarte, Amador C. Caballero, Marina Villegas, Ana Valle-Noguera, Aranzazu Cruz-Adalia, Marco Peiteado

**Affiliations:** 1 Department of Electroceramics, Instituto de Cerámica y Vidrio—CSIC, Kelsen 5, 28049 Madrid, Spain; jardiel@icv.csic.es (T.J.); erica.cordero-oyonarte@ensicaen.fr (E.C.-O.); amador@icv.csic.es (A.C.C.); marina.villegas@csic.es (M.V.); 2 Department of Immunology, School of Medicine, Universidad Complutense de Madrid, 12 de Octubre Health Research Institute (imas12), 28040 Madrid, Spain; avalle04@ucm.es (A.V.-N.); arancruz@ucm.es (A.C.-A.)

**Keywords:** optical bioimaging, fluorescent labels, ceramic composites, phosphorescent inorganics, long persistent luminescence, cellular uptake

## Abstract

In recent decades, the demand for biomedical imaging tools has grown very rapidly as a key feature for biomedical research and diagnostic applications. Particularly, fluorescence imaging has gained increased attention as a non-invasive, inexpensive technique that allows real-time imaging. However, tissue auto-fluorescence under external illumination, together with a weak tissue penetration of low wavelength excitation light, largely restricts the application of the technique. Accordingly, new types of fluorescent labels are currently being investigated and, in this search, phosphorescent nanoparticles promise great potential, as they combine the interesting size-dependent properties of nanoscale materials with a long-lasting phosphorescence-type emission that allows optical imaging well after excitation (so avoiding autofluorescence). In this work, core-shell structures consisting of SrAlO:Eu,Dy luminescent cores encapsulated within a biocompatible silica shell were prepared, showing a green persistent phosphorescence with an afterglow time of more than 1000 s. A high-energy ball milling procedure was used to reduce the size of the starting phosphors to a size suitable for cellular uptake, while the silica coating was produced by a reverse micelle methodology that eventually allows the excitation and emission light to pass efficiently through the shell. Confocal fluorescence microscopy using HeLa cancer cells confirmed the potential of the all-ceramic composites produced as feasible labels for in vitro optical imaging.

## 1. Introduction

In the last decade, nanomedicine has established as an area with great potential to address current problems and challenges related to the diagnosis and treatment of diseases, such as cancer, which is one of the top 10 leading causes of death in the world [1,2,3,4]. As it has been demonstrated, the survival rate for cancer improves by early diagnosis, and hence there is great need for new screening strategies and diagnostic methodologies capable of providing a faster and more effective detection of the disease [5,6,7]. In this context, molecular imaging plays a key role in personalized and targeted medicine [8] and, particularly, imaging modalities such as optical imaging (including fluorescence imaging), positron emission tomography (PET), and single-photon emission computed tomography (SPECT) have gathered considerable research interest for cancer diagnosis [9]. This is mainly due to the availability of a wide selection of molecules, with adequate properties, to provide a good signal that can be exploited to image a variety of cancers [10]. Among these techniques, fluorescence imaging reveals some clear-cut advantages, including superior sensitivity, low energy radiation, the capacity to monitor multiple independent optical biomarker reporters simultaneously (multiplexing), and a relatively simple imaging hardware [11,12,13]. Fluorescence techniques applied to date employ a number of well-established molecules further functionalized to target cancer specifically and can be also used to track and evaluate the efficiency of the drug release [14,15]. The vast majority of existing fluorescent labels applied in optical imaging are based on organic dyes [16]. However, these fluorophore-based molecular systems have their disadvantages: even the best fluorophores suffer from relatively low signals compared to enzymatic systems, they are prone to photobleaching in singleplex assays and they generally have wide emission spectra, which is technically limiting for multiplexing assays. Seeking to avoid these problems, the so-called quantum dots, fluorescent nanomaterials based on semiconductors, were developed [17]. However, the monodispersity of QDs must be ensured with rigorous synthesis qualifications based on the quantum confinement-induced size-dependent emissions. Moreover, hydrophobic QDs require further modification to achieve solubility in water. These two factors raise the cost of the QD-based bioprobes. Additionally, most available quantum dots contain cadmium, a toxic metal, which limits the potential for use in live humans/animals and which is also highly regulated in several countries due to its severe environmental hazard. Alternatively, inorganic luminescent nanoparticles based on suitably doped oxide-based matrices can provide probes with strong luminescence and yet have low toxicity [18,19,20]. Compared to current organic dyes, these inorganic cores possess higher photostability upon continuous excitation, always maintaining narrow emission maxima. Moreover, their size similarity to biomolecules ensures their potential for the investigation of biological events [13,21]. In this context, it would be of particular interest if, in addition to fluorescence, the inorganic nanoparticles could also show persistent luminescence, as this would allow for a time-gated imaging approach; for biological samples, the target fluorescent signal often suffers from interference with short-lived background fluorescence (reducing the target-to-background ratio), but this could be effectively eliminated using phosphorescent target signals with live times in the range of microseconds to thousands of seconds [22]. Inorganic phosphors with a long-lasting phosphorescence have been known for years and are typically composed of a host matrix (glass or ceramic) and two types of dopant ions: the luminescent centers (activators) and the traps. Phosphorescence is physically controlled by the traps’ concentration and their depth in the host material, generated by the deformation of the host (defects) and/or the presence of some impurities. Some typical sulphides (ZnS, CaS) were the first host materials for persistent phosphorescence, but they are chemically unstable and easily reacted with moisture to form hazardous hydrogen sulphide gas [23]. Phosphates, rare earth oxides, and oxysulfides can also be used as the host for long persistent phosphors but they cannot be charged by visible light, hence limiting their applications [24]. Alternative hosts such as yttrium- and gadolinium-based garnets, perovskite type niobates and titanates, or zinc gallates and germanates are currently under investigation, but so far the most efficient persistent luminescence has been observed for alkaline-earth aluminate and silicate hosts such as SrAl_2_O_4_, Sr_4_Al_14_O_25_, CaAl_2_O_4_, Sr_3_MgSi_2_O_8_, or Ca_2_Mg_2_Si_2_O_7_ [25]. This is mainly because defects are easily created in these hosts due to the charge compensation and cation disorder mechanisms [24]; in addition, they exhibit wide band gaps, allowing deep traps to be created and further increasing the persistent lifetime [26,27]. As for the dopants, transition metals or rare earth elements are employed as activators and traps. For example, transition metals with a 3d state such as Mn^2+^, Mn^4+^, and Ti^4+^ or rare earth ions with a 5d state such as Ce^3+^, Eu^2+^, Eu^3+^, and Tb^3+^ are good candidates for activator luminescent centers; among them, the Eu^2+^ is the most famous isolated phosphorescent center due to its half shell-filled characteristic, with an emission wavelength from 4f^6^5d^1^ to 4f^7^ that changes from host to host, leading to wide coverage of the spectrum [28]. On the other hand, a co-dopant is added to produce defect-related trapping centers, and, for example, trivalent lanthanide ions such as Nd^3+^ or Dy^3+^ can greatly enhance the trap populations in the host [24,26]. Accordingly, in the last few years more than 200 combinations of host materials and ions have been depicted, of which about 20% are focused around divalent europium (Eu^2+^) and other codopants [29]. In this wide range of alternatives, strontium aluminates such as SrAl_2_O_4_:Eu,Dy and its derivative Sr_4_Al_14_O_25_:Eu,Dy are among the best performing persistent luminescent phosphors in terms of afterglow time and intensity. These materials are already in commercial use for several applications, including emergency signage, textile printing, dials and displays, or photovoltaics, while their use in medical applications as imaging labels is still at an early stage of research [29,30,31].

In this context, this work aims to advance the study of these phosphorescent strontium aluminates as potential bioimaging probes. One of the main challenges is to ensure an effective binding of the luminescent particles to the biological targets without affecting their optical performance. In doing so, it is first necessary to encapsulate the inorganic cores with a biocompatible shell which, being optically transparent, also facilitates subsequent internalization of the probes into the cells. Specifically, we used a commercial Eu^2+^,Dy^3+^-doped Sr_4_Al_14_O_25_ phosphor material as the luminescent core and mesoporous silica to form the biocompatible shell. A high-energy ball milling procedure is used to reduce the size of commercial phosphors to a size suitable for cellular uptake, while the homogeneous silica coating is obtained by a reverse micelle routine. In the obtained composites, the silica shield not only provides biocompatibility to the probes but also protects the luminescent cores from lixiviation, and detection/degradation by the immune system. The light transparency of the shell is regulated by its crystallinity, eventually allowing the excitation and emission light to pass through efficiently. With this in mind, the synthetic conditions were optimized to achieve highly homogeneous core-shell structures with long-lasting phosphorescence properties. The performance of the all-ceramic composite labels was evaluated in a human cervix carcinoma cell line (HeLa), confirming their suitability for in vitro imaging.

## 2. Results and Discussion

**Figure 1:**
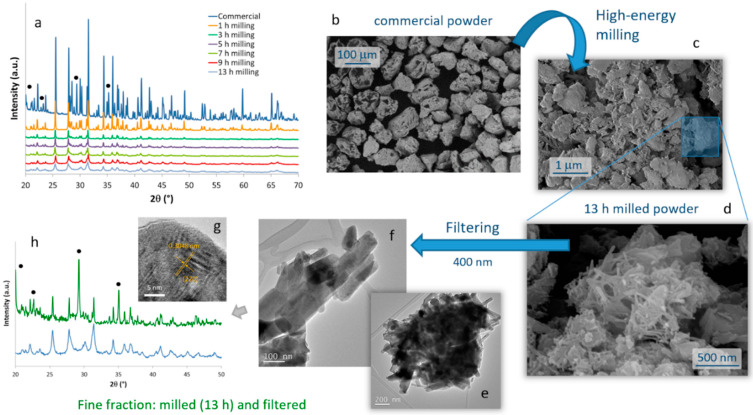
Processing steps to obtain the SrAlO:Eu,Dy nanoparticles. (**a**) Evolution of the high-energy milling process of the commercial powder as followed by XRD. Black circles indicate the maxima of the SrAl_2_O_4_ phase, minority at this point, all other peaks correspond to the Sr_4_Al_14_O_25_ phase. (**b**–**d**) SEM images at different times of the mechanosynthesis stage and (**e**–**g**) nanoparticles obtained after filtration of the milled powder for 13 h. (**h**) XRD of the 13 h milled (blue pattern) and filtered (green) powder indicating that the major phase is now SrAl_2_O_4_ (black circles).

The evolution of the crystalline phases present in the SrAlO:Eu,Dy commercial powder during the high-energy milling procedure was monitored by XRD on samples milled during different times, Figure 1a. The diffractogram corresponding to the starting powder shows sharp diffraction maxima which, although slightly displaced due to the rare earth doping, can be ascribed to two different crystalline phases: the orthorhombic Sr_4_Al_14_O_25_ (ICDD entry: 96-600-0236) as the main phase and, to a lesser extent, the monoclinic SrAl_2_O_4_ phase (ICDD: 00-034-0379). The presence of these two phases is the usual picture, and both are effectively luminescent and responsible for the long-lasting phosphorescent properties [32,33]. After 3 h of high-energy milling, the sharp peaks are replaced by a few broad signals that largely correspond to the Sr_4_Al_14_O_25_ doped phase. The observed broadening denotes amorphization and implies a rapid reduction in the powder particle size. As the milling time increases, the diffraction maxima broaden and the particles in the powder become smaller, until for a given milling time no effective reduction in crystal size is achieved anymore. On the contrary, beyond this time, the mechanochemical activation can lead to excessive amorphization of the crystalline phase, generating multiple defects on the particle surfaces that could eventually bring a loss of luminescent properties. Moreover, the local coordination environment offered to the dopants in the strontium aluminate hosts is highly dependent on the crystallinity and lattice microstructure, and it could even happen that the energy invested in particle size reduction would also oxidize some Eu^2+^ ions into inactive Eu^3+^ in the host lattice (with a consequent loss of optical performance [32]). With this in mind, different tests were conducted and finally DLS measurements of particle size led us to set an optimum milling time of 13 h.

The microstructural evolution of the milled samples was monitored by scanning electron microscopy (FESEM). Micrographs in Figure 1b,c correspond to the commercial powder and the material resulting from the 13 h of milling, respectively. They confirm the drastic decrease in particle size caused by the mechanochemical process, going from an average particle size of almost 100 microns in the commercial powder, to particles of the order of one micron and below in the milled sample. Two types of particles actually compose the milled material. ‘Large’ particles of ca. 1 micron in size (which to some extent retain the original shape of the particles in the commercial powder), together with a considerable fraction of fine particles with an elongated morphology and dimensions already in the nanometric scale were observed, Figure 1d. The latter are of particular interest for the preparation of the composite bioprobes, and so they were extracted by filtration through a 400 nm mesh. The result of this separation is shown in the TEM images in Figure 1e–g, where it can be seen that the elongated particles have dimensions of about 100–400 nm in length and less than 50 nm in diameter. The EDS analysis confirmed the presence of Sr, Al, and the two RE dopants in these crystals without any trace of other impurities, but the XRD measurements unveiled an interesting result. As can be seen in Figure 1h, in this powder filtered from the 13 h milled sample, the Sr_4_Al_14_O_25_ orthorhombic phase became a minority, while the SrAl_2_O_4_ phase and specifically its hexagonal polymorph (ICDD entry: 00-031-1336) is now the majority compound. Indeed, this hexagonal symmetry agrees well with the elongated morphology seen in most of the filtered nanoparticles. The magnified HRTEM image of one of these nanoparticles (Figure 1g) authorizes this attribution, as it reveals an interplanar distance of 3.048 Å that matches the (220) crystallographic plane (2θ = 29°) of a doped SrAl_2_O_4_ phase. From the point of view of optical properties, this ‘switch’ between majority phases is not expected to represent a serious problem for the intended bioimaging purposes, since as indicated the strontium aluminate compounds and their various polymorphs all render strong luminescence [34]. However, it is still a remarkable finding since under normal conditions the hexagonal phase of SrAl_2_O_4_ is only stable above 675 °C [35]. We may explain it on the fact that the high energy provided during the mechanochemical process not only reduces the particle size, but also catalyzes (mechanosynthesis activation) the monoclinic–hexagonal phase transition of SrAl_2_O_4_, enabling the stabilization of the hexagonal symmetry at room temperature. Subsequent filtering of the milled powder discards the larger particles corresponding mostly to the Sr_4_Al_14_O_25_ phase and mainly collects the newly transformed hexagonal SrAl_2_O_4_. Scherrer calculations using the width of the SrAl_2_O_4_ phase maximum at 2θ~29° yield a crystallite size of 44 nm for this (milled and filtered) fine fraction of the powder.

**Figure 2:**
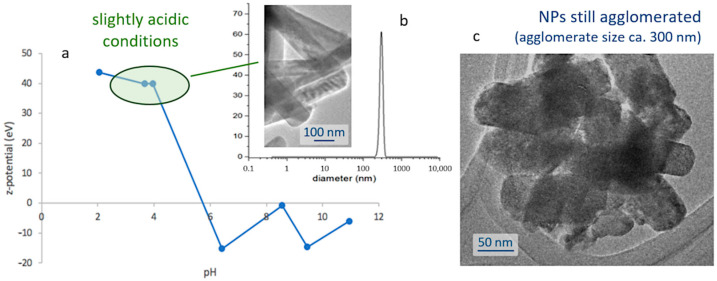
(**a**) ζ–potential measurements of SrAlO:Eu,Dy nanoparticle suspensions as a function of pH conditions. (**b**) Particle size (DLS) of the suspension produced at pH 4. (**c**) TEM image showing the agglomeration tendency of the suspended NPs.

The next step towards the assembly of the bioprobes involves encapsulating the obtained nanoparticles within a silica shell; thus, protecting them from degradation in the cellular environment, while ensuring the biocompatibility of the biomarkers. This first requires the preparation of stable and well-dispersed suspensions of the nanoparticles, and so their natural tendency to agglomerate (Figure 1e) and subsequently flocculate, shall be overcome. To that end, a systematic study of the surface characteristics of those fine particles was conducted by preparing a series of pattern suspensions and measuring the corresponding ζ-potential. The strontium aluminate phosphors are found to be sensitive to water [36,37], so in order to avoid the hydrolysis of the phosphor particles the dispersion was conducted in ethanol and, hence, the pH values are just indicative as corresponding to [H^+^] in ethanol medium. Depicted in Figure 2a, the results of this study indicate that the degree of agglomeration of the luminescent nuclei decreases in acidic media (higher ζ-potential values), meaning that it will be necessary to work in slightly acidic conditions to produce the suspensions. In particular, the most stable scenarios were obtained at pH 4 and for concentrations of 1 mg/mL, yielding a mono-modal dispersion centered around 300 nm, Figure 2b; it should be noted, however, that this value is slightly larger than the average size of the individual particles, and actually indicates that despite the use of a dispersing agent (IGEPAL, see Section 3) there is still some agglomeration between them, as also confirmed in the transmission electron microscope, Figure 2c.

**Figure 3:**
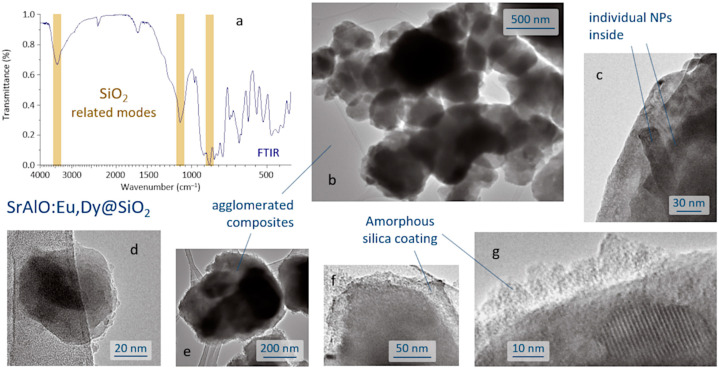
Production of SrAlO:Eu,Dy@SiO_2_ composites by a reverse micelle protocol. (**a**) FT-IR spectrum of the sample obtained after the coating process. (**b**–**g**) TEM images of the composites showing different details of the core-shell structure.

The SrAlO:Eu,Dy@SiO_2_ core-shell composites were produced by a reverse micelle protocol (cyclohexane, acidic conditions) and using TEOS as the silica source. The FTIR spectrum of the sample obtained after the coating routine is displayed in Figure 3a and shows the presence of a broad band near 3440 cm^−1^ corresponding to the –OH stretching vibration of the incomplete condensation of the silanol group (SiOH). The vibration’s peaks belonging to the SiO_2_ groups are assigned to the asymmetric and symmetric stretching modes observed at 1102 and 844 cm^−1^, associated with the O–Si–O and Si–O–Si symmetric stretching, and confirm the formation of the SiO_4_ network of the SiO_2_ shell structure [38,39]. In addition, the spectrum exhibits a peak at ~2400 cm^−1^, assigned to the stretching of C=O bond, and a strong peak at ~1630 cm^−1^ assigned to the C–O–C anti-symmetric bonds, these two actually resulting from the adsorption of atmospheric CO_2_ on the sample surface. Metal oxygen stretching frequencies in the range 700–1000 cm^−1^ are associated with the vibrations of Al–O and Sr–O–Al bonds. The anti-symmetric stretching bonds between 500 and 650 cm^−1^ are attributed to the Sr–O vibrations, whereas the symmetric bonding of O–Al–O shows up at around 450 cm^−1^ [40]. The microstructural characterization of the as-prepared composites was performed by TEM, Figure 3b–g. As can be seen, the reverse micelle process allows effective coating of the luminescent nuclei, each core being composed of the agglomeration of several individual nanoparticles, Figure 3c. The estimated size of the core-shell composites is about 100–200 nm, Figure 3d, although they also tend to agglomerate, yielding submicron clusters of around 400–500 nm in size, Figure 3e. On the other hand, the shell, which is amorphous at this stage of the process, displays an average thickness of less than 20 nm, Figure 3f,g, which in principle could be adequate to enable efficient excitation and emission of light through its framework [13].

**Figure 4:**
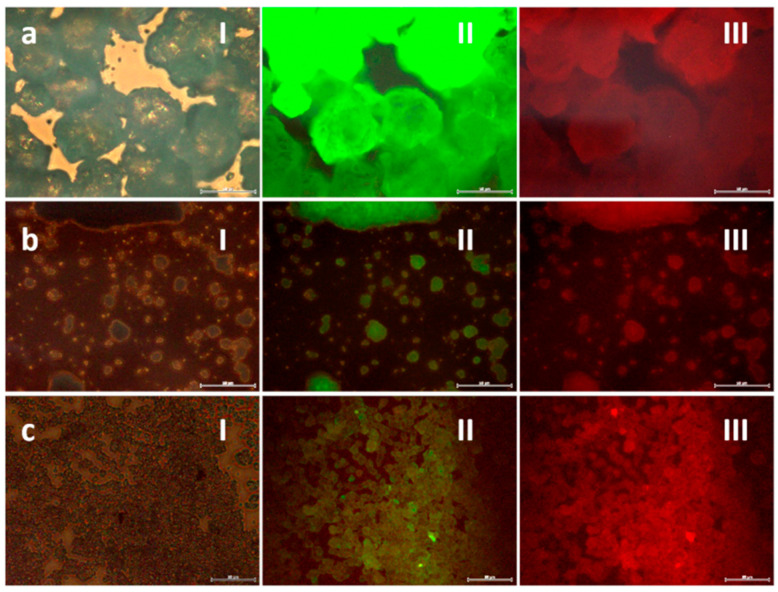
Evolution of the luminescent intensity as followed by fluorescence microscopy. (**a**) Commercial SrAlO:Eu,Dy powder (scale bar 50 μm), (**b**) 13 h milled powder (scale bar 50 μm), (**c**) as-prepared SrAlO:Eu,Dy@SiO_2_ composites (scale bar 20 μm). Measurements performed in (**I**) bright field channel; (**II**) green channel, λ_em_ = 500–550 nm; and (**III**) red channel, λ_em_ = 570–700 nm; λ_ex_ = 405 nm.

This was corroborated by fluorescence microscopy and the results are shown in Figure 4. As can be seen, this particular phosphor composition produces both green and red emissions, and initially there is a reduction in luminescence intensity as the particle size decreases (milling stage) that also extends to the subsequent coating of the milled nanoparticles. This is because during milling, part of the energy provided to the system causes the formation of electronic defects on the surface of the luminescent cores. The presence of these defects destabilizes the energy levels locally, altering the decay transition of the photoexcited electrons responsible for the fluorescent emission (and the intersystem crossing processes responsible for the phosphorescence). The detrimental effect of surface defects on luminescence intensity can be mitigated by the silica shielding, although it is first necessary to thermally consolidate the composites. This last annealing step crystallizes the SiO_2_ shell, promoting the closure of its internal and interconnected porosity and favoring a firmer isolation of the luminescent cores from the medium. In addition, moderate heating can promote a partial re-crystallization of the luminescent nuclei, further contributing to the elimination of the aforementioned surface defects. Accordingly, a low temperature treatment at 400 °C for 30 min was applied to the composites (higher temperatures might cause unwanted interdiffusion processes).

**Figure 5:**
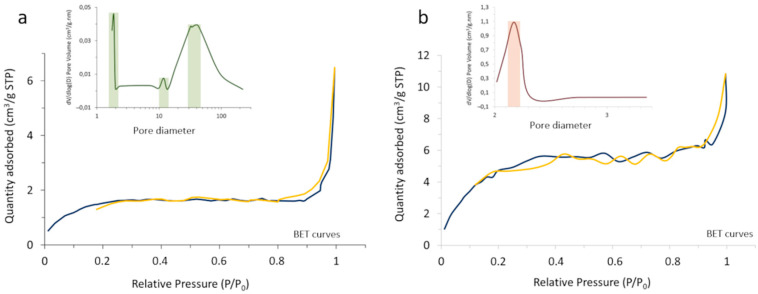
N_2_ adsorption (blue)–desorption (yellow) BET isotherms and pore size distribution of (**a**) the as-prepared SrAlO:Eu,Dy@SiO_2_ powder and (**b**) the powder calcined at 400 °C/30 min.

The outcome of the process was first characterized by BET and N_2_ adsorption–desorption measurements, monitoring the change in the surface characteristics and porosity of the core-shell composites with the heat treatment (Figure 5). In particular, it was observed that the specific surface area of the powder increases from 5.62 m^2^/g in the as-prepared composite to 19.83 m^2^/g in the calcined system, which should be understood in the context of changing from an amorphous coating with a poorly defined, blurred surface to a consolidated scenario where the coating layer has crystallized and gives rise to well-defined surfaces. On the other hand, the porosity evolved satisfactorily from a tri-modal distribution in the as-prepared material (with pore sizes centered at ca. 2, 12, and 35 nm), to a mono-modal distribution centered at ~2 nm in the calcined sample. Such sealing effectively encapsulates the luminescent nuclei, which as mentioned is a key point to avoid interactions with the surrounding media and/or possible leaching.

**Figure 6:**
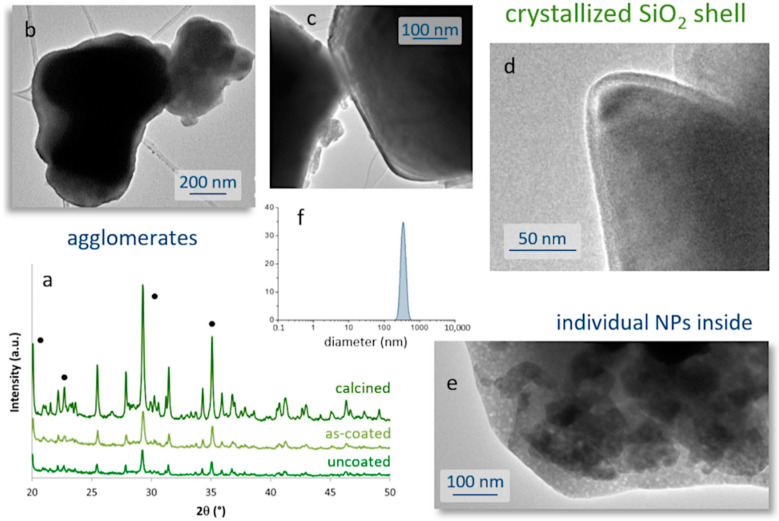
Crystallization of the silica shell to consolidate the SrAlO:Eu,Dy@SiO_2_ composites. (**a**) Evolution of the crystalline phases after coating and subsequent calcination at 400 °C, as followed by XRD. Black circles indicate the maxima of the SrAl_2_O_4_ phase, all other peaks correspond to the Sr_4_Al_14_O_25_ phase. (**b**–**e**) TEM images of the calcined core-shell composites. (**f**) Particle size distribution after crystallization.

The consolidation treatment was also examined using FTIR, XRD, DLS, and TEM analyses. The FTIR spectrum of the fired sample showed no significant changes with respect to that of the pre-heated composite. Differences were observed in the XRD analysis, with the consolidation process leading to sharper and narrower maxima in the recorded diffractogram, Figure 6a. The detected maxima can be assigned to the hexagonal SrAl_2_O_4_ phase, which remains the major phase after heat treatment, and to the orthorhombic phase Sr_4_Al_14_O_25_. The higher intensity of these peaks masks the detection of the crystallized silica shell, but in any case, their pronounced narrowing does indicate an overall increase in the composite’s crystallinity. In particular, the evolution of crystallite size, as estimated from the Scherrer equation (width of the SrAl_2_O_4_ maximum at 2θ~29°), indicates that the crystallites of this hexagonal phase undergo an increase in size from 44 nm in the uncoated powder to 48 nm after heating the composite material, corroborating the improvement in crystallinity. As mentioned before, this re-crystallization of the luminescent nuclei is very propitious as it eliminates surface defects and improves their optical response. TEM images also confirm the effects of the consolidation stage, resulting in composites with sharper and more defined surfaces, as shown in Figure 6b–e. As with the parent nanoparticles, the composites also show a strong tendency to agglomerate. DLS analyses indicate that a mono-modal size distribution is preserved after the coating (Figure 6f), although the average size is slightly shifted to larger values: 340 nm for the consolidated composites versus 300 nm of the (agglomerated) uncoated nanoparticles (Figure 2b). Actually, the average thickness of the SiO_2_ layer after crystallization remains in the range of 10–20 nm, and in some regions it is even less than 10 nm (Figure 6c,d). On the other hand, the recrystallization of the luminescent nuclei within the composite core does not entail a loss of their nanometric dimensions, as shown in Figure 6e.

**Figure 7:**
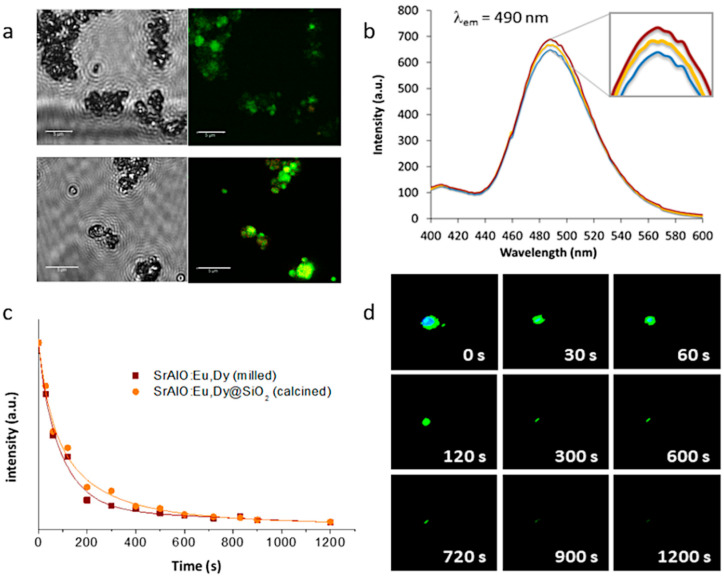
Optical performance of the SrAlO:Eu,Dy@SiO_2_ core-shell composites. (**a**) Confocal fluorescence micrographs corresponding to the as-prepared composites (top images) and after the calcination process at 400 °C (bottom images); measurements were performed in bright-field and green channel (λ_em_ = 500–550 nm); λ_ex_ = 405 nm. Scale bar: 5 μm. (**b**) Fluorescence emission spectra of the milled powder (red line), the as-prepared composites (blue line) and the consolidated material (yellow line), upon excitation wavelength at 345 nm. (**c**) Afterglow curves for the persistent luminescence of the uncoated and SiO_2_-coated materials (λ_exc._ = 345 nm, λ_em._ = 490 nm). (**d**) Time-lapse frames showing green long-lasting phosphorescence in the consolidated composites (λ_em_ = 510 nm).

Eventually, this configuration, in which several optically active nanoparticles are together encapsulated within an (optically) transparent SiO_2_ shell, could even lead to a higher number of emitted photons per unit area, which would result in a higher luminescent intensity and a longer persistency. This was first investigated by fluorescence microscopy, this time using a laser scanning confocal imaging device that allows better identification of the fluorescent probes. The resulting confocal fluorescence micrographs (bright-field channel) indicate an intense green luminescence for both the as-prepared SrAlO:Eu,Dy@SiO_2_ core-shell units and the calcined composites, Figure 7a; however, they also show that such annealing at 400 °C to consolidate the SiO_2_ shell, by improving the overall crystallinity of the luminescent system, eventually results in a notable enhancement of their fluorescence intensity. The corresponding fluorescence emission spectra of the nanocomposites before and after heat treatment are shown in Figure 7b, showing the presence of a strong emission band around at 490 nm which is attributed to the spin-allowed 4f^6^5d^1^ → 4f^7^ transition in the Eu^2+^ centers (green emission) [41]. As can be seen, the coating of the milled powders with the silica shell produces a decrease in fluorescence intensity related to the initial shielding of the luminescent nuclei with an amorphous SiO_2_ layer. Subsequent heat treatment causes this layer to crystallize, eliminating surface defects and stabilizing the energy levels of the composite. The increase in crystallinity also affects the internal structure of the materials, specifically by stabilizing the atomic positions and, therefore, the electronic structure of Eu^2+^. This actually means that the electronic transitions are more defined and favored, hence, the observed increase in fluorescence with an intensity that practically recovers that of the milled sample. Finally, the phosphorescence afterglow properties of the annealed SrAlO:Eu,Dy@SiO_2_ composites were evaluated. Figure 7c shows the decay curves for the uncoated milled powder as well as for the composite material with the SiO_2_ shell (and already calcined), showing in both cases a persistent response over time. The observed phosphorescence is slightly more intense for the composite sample, confirming the beneficial role of the silica shielding in mitigating the presence of defects on the surface of the milled powder. On the other hand, Figure 7d shows a series of video-recorded frames captured at different times after irradiating the samples with UV light. As observed, the prepared core-shell systems exhibit a long-lived phosphorescence of up to 15 min which optically validates them for a time-gated imaging approach. The newly synthesized materials were subsequently tested for their uptake in living cells.

### Cellular Uptake (In Vitro) Studies

**Figure 8:**
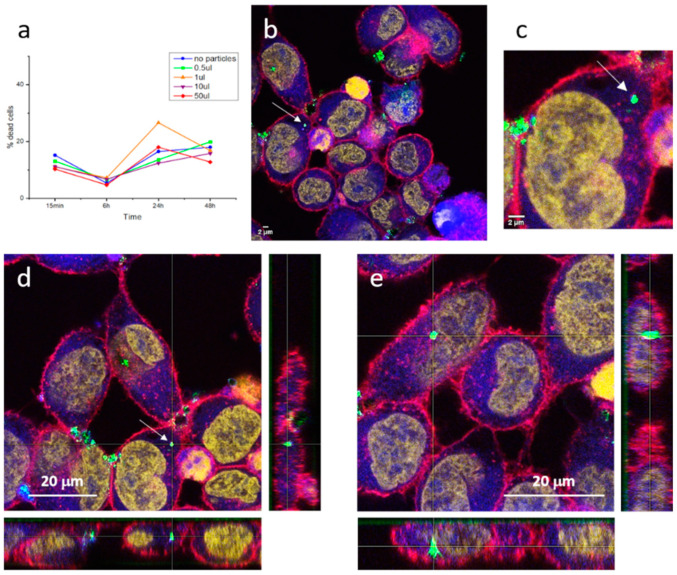
(**a**) Flow cytometry analysis of cell cytotoxicity after 15 min, 6 h, 24 h, and 48 h of exposure of HeLa cells to different concentrations of SrAlO:Eu,Dy@SiO_2_ powder 400 °C/30 min. (**b**–**e**) Confocal images of HeLa cells incubated with the SrAlO:Eu,Dy@SiO_2_ composites at 100 µg/mL for 30 min. After PBS washes, cells were stained with 0.2 µM calcein for 30 min at 37 °C + 5 µg/mL WGA for 5 min at RT + 1 µM TO-PRO for 30 min at RT. Measurements were performed with Laser line 488 nm/HyD 497–555 nm (ex/em), Laser line 550 nm/HyD 559–630 nm (ex/em), Laser line 633nm/HyD 648–736 nm (ex/em), and Laser line 561nm/PMT 550–573 nm (ex/detection reflection). The following reference colors are used: green: SrAlO:Eu,Dy@SiO_2_ composites; red: membrane; blue: cytoplasm; yellow: cellular nucleus. White arrow indicates a composite (or small cluster of composites) inside the HeLa cell.

Standard flow cytometry assays of HeLa cells incubated with SrAlO:Eu,Dy@SiO_2_ composite suspensions were first performed in order to investigate the cellular viability of the candidate biomarkers. As described in the Section 3, the in vitro protocol includes incubation with different concentrations of the composites and subsequent monitoring of the percentage of dead cells over time. Figure 8a depicts the result of these cell viability studies and clearly denotes that the core-shell particles do not kill the cells, since similar mortality percentages are obtained both in the presence and the absence of the composites (detected fluctuations being attributed to the death of the cell line in culture). In addition, after two days of incubation (48 h) none of the cell cultures incubated with the composites produced an increase in cell mortality, remaining in the same percentages as the reference culture without particles. This confirms that the encapsulation of the luminescent nuclei with the silica shell is effective and authorizes the obtained composite probes as innocuous and biocompatible.

Subsequently, laser scanning confocal and epifluorescence imaging techniques were used to examine the morphology and fluorescent distribution of the fluorescent probes in cells, Figure 8b,c. The images obtained first show that the phosphorescent composites maintain their luminescent intensity in the cell medium, where they are readily detected by the confocal microscope (strong green signal). The morphology of cells was as expected, cells size is approximately 20 µm and irregularly rounded with microvilli present at the surface, so there is no sign of membrane damage caused by the inoculated particles [42]. As for cellular uptake, interesting results are also obtained. On one hand, the composites tend to accumulate to form large agglomerates that, due to their size, cannot enter the cells and, thus, concentrate in the regions in between cells. This situation, common when dealing with nanoparticulate systems, already appeared when obtaining the milled nanoparticles and in the preparation of the composites themselves, although the incorporation of surfactant additives made it possible to obtain stable dispersions in suspension. In the cellular medium, the same type of dispersants cannot be used, and tendency to agglomeration again shows up. This is not a minor problem and further studies would be necessary to properly functionalize the surface of the composites and avoid agglomeration. However, on the other hand, the recorded images also indicate that when the composites are not agglomerated in large clumps, the cells uptake them effectively. This is the case of the composite (or small cluster of composites) indicated with an arrow in Figure 8b and magnified in Figure 8c. As can be seen, the retrieved fluorescent signal indicates the presence of this composite in the cell cytoplasm. To discard any possible optical effect (i.e., the composite being located above the cell), z-stacking analyses confirmed its presence inside the HeLa cell, Figure 8d. The same tracking of another composite detected inside the HeLa cells is shown in Figure 8e, again confirming effective capture by the cancerous entities. Overall, a systematic study of the inoculated cells revealed more patterns similar to those in Figure 8d,e (for more examples, see Figure S1 in Supplementary Info), demonstrating that, as long as they are not agglomerated, the produced SrAlO:Eu,Dy@SiO_2_ phosphorescent bioprobes are readily incorporated into the cells, which confirms them as promising biomarkers for in vitro optical imaging.

## 3. Materials and Methods

### 3.1. Preparation of the SrAlO:Eu,Dy Core Particles

Commercial micron powder of nominal composition Sr_3.89_Eu_0.06_Dy_0.10_Al_14_O_25_ (Sigma-Aldrich, purity: 99% trace metal basis) was chosen as the luminescent core material due to its long-persistent luminescence properties. However, in order to use these inorganic particles as imaging agents that can properly reach the cells, their size must be substantially reduced and fall well into the submicronic or even nanometric range. This was accomplished by applying a high-energy milling procedure in a tungsten carbide (WC) planetary mill (Retsch, Haan, Germany), using 2 cm diameter WC milling balls in alcoholic medium. The grinding vessels were rotated at 300 rpm for different milling times. The optimum milling time was chosen on the basis of the X-ray diffraction and SEM characterization results. The powder obtained was re-suspended in ethanol and filtered through a 400 nm polymeric mesh coupled within a conical centrifuge tube (Falcon, Sigma-Aldrich) to obtain a fine fraction of particles. Time in the centrifuge did not exceed 2 min, after which the powder not passing through the mesh was discarded.

### 3.2. Assembly of the SrAlO:Eu,Dy@SiO_2_ Composites

The encapsulation of the SrAlO:Eu,Dy with a silica shell was accomplished using a reverse micelle routine in its acidic variant [10]. In a typical procedure, 0.1 g of the strontium aluminate nanoparticles were suspended in cyclohexane (1 mg/mL) in a round bottom flask. A dispersant, IGEPAL CA-520 (12.5 g, Sigma-Aldrich), was also incorporated to prevent agglomeration of the suspended particles, and the whole mixture was kept under stirring until a stable suspension was observed: no precipitation upon ceasing stirring. In a second step, acetic acid was added until pH 4 to form the reverse micro-emulsion and subsequently 1.925 mL of tetraethyl orthosilicate (TEOS, Sigma-Aldrich) were incorporated. The suspension was gently stirred for 16 h to complete the polymerization of the silica precursor and the precipitate obtained was finally separated by filtering and centrifugation. As-prepared composites were consolidated by mild heating at 400 °C/30 min in air.

### 3.3. Characterization of the Synthesized Nanoparticles and Composites

The analyses of the crystalline structure and phase identification were performed by X-ray diffraction (XRD Bruker D8 ADVANCE, Bruker, Billerica, MA, USA) with a monochromatized source of Cu-Kα1 radiation (λ = 1.5406 nm) at 1.6 kW (40 KV, 40 mA); samples were prepared by placing a drop of a concentrated ethanol dispersion of particles onto a single crystal silicon plate. The crystallite size was estimated from the full width at half maximum of a specific diffraction peak using the Sherrer’s equation. Field emission scanning electron microscopy (FESEM) was employed to characterize the main microstructural features of the milled particles and composites; a cold FESEM S-4700 microscope (Hitachi, Chiyoda, Tokio, Japan) was used for that purpose. Further investigation of the particles’ morphology and size was conducted by high-resolution transmission electron microscopy (HRTEM) using a JEOL 2100F transmission electron microscope (TEM/STEM; Akishima, Tokio, Japan) operating at 200 kV. The microscope is equipped with a field emission electron gun (point resolution 0.19 nm) and coupled to a INCA X-Sight energy dispersive X-ray (EDX) spectrometer for elemental analysis (Oxford Instruments); samples were prepared by placing a drop of a dilute ethanol dispersion of nanoparticles onto a 300 mesh carbon-coated copper grid and evaporating immediately at 60 °C. Particle size distribution measurements of the phosphor powders was completed by means of dynamic light scattering (DLS) on a Malvern Zetasizer Nano ZS instrument (Malvern Panalytical, Malvern, UK). The same apparatus was used to evaluate the dispersion and stability of the nanoparticle suspensions, measuring the ζ-potential at 25 °C (samples were suspended in 1 mL absolute ethanol and placed in Zetasizer disposable cuvettes). BET surface area and pore size distribution measurements (N_2_ adsorption/desorption isotherms) were undertaken using a Quantochrome surface analyzer (Bounton Beach, FL, USA). Infrared spectroscopy FTIR (IR-Prestige-21, Perkin-Elmer, Walthan, MA, USA) was also used to monitor the assembly of the core-shell composites. Fluorescence microscopy images were taken on a Trinocular Optical Fluorescence Microscope (Olympus CX, Tokio, Japan). The fluorescence emission spectra of the phosphor materials were recorded over the 400–600 nm wavelength range on a Varian Cary Eclipse Fluorescence Spectrophotometer (Agilent, Santa Clara, CA, USA), using a xenon arc lamp as the excitation source (λ_exc._ = 345 nm). The same machine was used for phosphorescence afterglow measurements, irradiating the samples for 2 min (λ_exc._ = 345 nm) and subsequently measuring the intensity decay with time (λ_em._ = 490 nm). Light emission with time was also video recorded (λ_em._ = 510 nm) and picture frames were taken at different intervals until no light was detected to the naked eye.

### 3.4. In Vitro Fluorescence Experiments

Single photon confocal fluorescence imaging was measured in a human cervix carcinoma (HeLa) cell line incubated with the SrAlO:Eu,Dy@SiO_2_ composites. Cells were kept in culture in DMEM glutamax (Gibco) supplemented with 10% FBS (Corning), 1% Penicilin/Streptomicin (Sigma-Aldrich), and 1% Nonessential Amino Acids (Gibco) in a flat-bottomed plate prior to the experiment (see below). Coverslips were treated with 0.01% Poly-L-Lysine (Sigma-Aldrich) and 200.000 cells were plated for 30 min. Once cells were adhered, 1 mL of culture media was added and kept in culture for 24 h in flat-bottomed 24-well plates. Composites suspensions were sonicated and vortexed thoroughly before adding 50 µL in each well. After 30 min of incubation, cells were washed three times with Phosphate Buffer Saline (PBS) and stained with 0.2 µM Calcein (Biotium, Fremont, CA, USA) during 30 min at 37 °C, and soon after they were washed with PBS and stained with 5 µg/mL Wheat Germ Agglutinin (WGA) (Thermo Fisher Scientific) during 5 min at room temperature (RT). Cells were then washed and fixed with 4% paraformaldehyde in PBS for 10 min, washed with PBS 1X, and permeabilized with 0.1% Triton X-100 in Tris Buffer Saline (TBS) for 5 min. Subsequently, cells were washed with PBS and stained with 1 µM TO-PRO^®^-3 stain (Thermo Fisher Scientific, Waltham, MA, USA) for 30 min at RT, washed three times with PBS 1X, and one time with distilled water. Finally, coverslips were mounted upside down with Mowiol (Sigma-Aldrich). The incubated cells were analyzed in the confocal multispectral Leica TCS SP8 system (Leica, Wetzlar, Germany) with a 3X STED module for super-resolution with HC PL APO CS2 63x/1.40 oil objective. Cells were detected with laser line 488 nm/Hybrid Detector (HyD) 497–555 nm (ex/em), laser line 550 nm/HyD 559–630 nm (ex/em), and laser line 633nm/HyD 648–736 nm (ex/em), whereas composites were detected using reflection contrast microscopy (RCM) with laser line 561nm/Photomultiplier Tube (PMT) 550–573 nm (ex/detection reflection). Images were processed with ImageJ software.

### 3.5. Cell Culture Preparation and MTT Assays Tests for IC50 Estimation

The HeLa cell line was grown according to standard serial passage protocols. In total, 100.000 cells were cultured in DMEM glutamax (Gibco, Waltham, MA, USA) supplemented with 10% FBS (Corning, New York, NY, USA), 1% Penicilin/Streptomicin (Sigma-Aldrich, St. Louis, MO, USA), and 1% Nonessential Amino Acids (Gibco) in flat-bottomed 24-well plates 24 h prior to the experiment. Composite suspensions were sonicated and vortexed thoroughly before adding into the cell culture. Then, 0.1, 1, 10, and 50 µL of composites suspensions were incubated with the cells for 15 min, 6 h, 24 h, and 48 h. After the different times, supernatant containing dead cells was collected and adherent cells were detached using trypsin (Lonza, Bend, OR, USA) and added to the same tube containing the supernatant. Cells were washed with PBS 1× and stained with 2.5 µg/mL of 7-aminoactinomicina D (7ADD) (BD Pharmingen, Franklin Lakes, NJ, USA) for 45 min at 4 °C. Cell viability was analyzed by fluorescence using a BD FACSCalibur flow cytometer (Becton Dickinson, Franklin Lakes, NJ, USA), due to the staining of non-viable cells by the 7ADD impermeant dye.

## 4. Conclusions

Phosphorescent materials can be excellent candidates for biomedical diagnostic applications as their long persistence luminescent response may enable time-gated imaging. In this work, the potential of a commercial RE-doped strontium aluminate phosphor material was evaluated by first bringing its particle size to the nanoscale range. A systematic mechanosynthesis process followed by selective filtration was carried out for this purpose. The obtained nanoparticles were subsequently encapsulated within a biocompatible silica shell that allows phosphorescent light to pass through (live times above 1000 s) and further protects the luminescent nuclei from lixiviation and detection (and degradation) by the immune system. In vitro tests confirmed successful uptake of the core-shell composites into cancerous HeLa cells, where they yielded a strong optical fluorescence. There is, however, a problem of agglomeration that needs to be improved in order to exploit the potential of these phosphors in optical imaging. Future work is planned in this direction, specifically addressing the synthesis of the phosphorescent nanomaterial by bottom-up strategies that can prevent the agglomeration of the nanoparticles from the very beginning of the core shell units’ preparation. With increased uptake, the diagnostic capabilities of these optical markers could also be extended to certain biomedical applications in vivo.

## Data Availability

Not applicable.

## Supplementary Materials

The following supporting information can be downloaded at: https://www.mdpi.com/article/10.3390/ijms23063410/s1.
