# Supplementary figures and images for: Biocompatible Probes Based on Rare-Earth Doped Strontium Aluminates with Long-Lasting Phosphorescent Properties for In Vitro Optical IMAGING
Source: Int J Mol Sci. 2022 Mar 21;23(6):3410. doi: 10.3390/ijms23063410 (PMC8954243; doi:10.3390/ijms23063410)

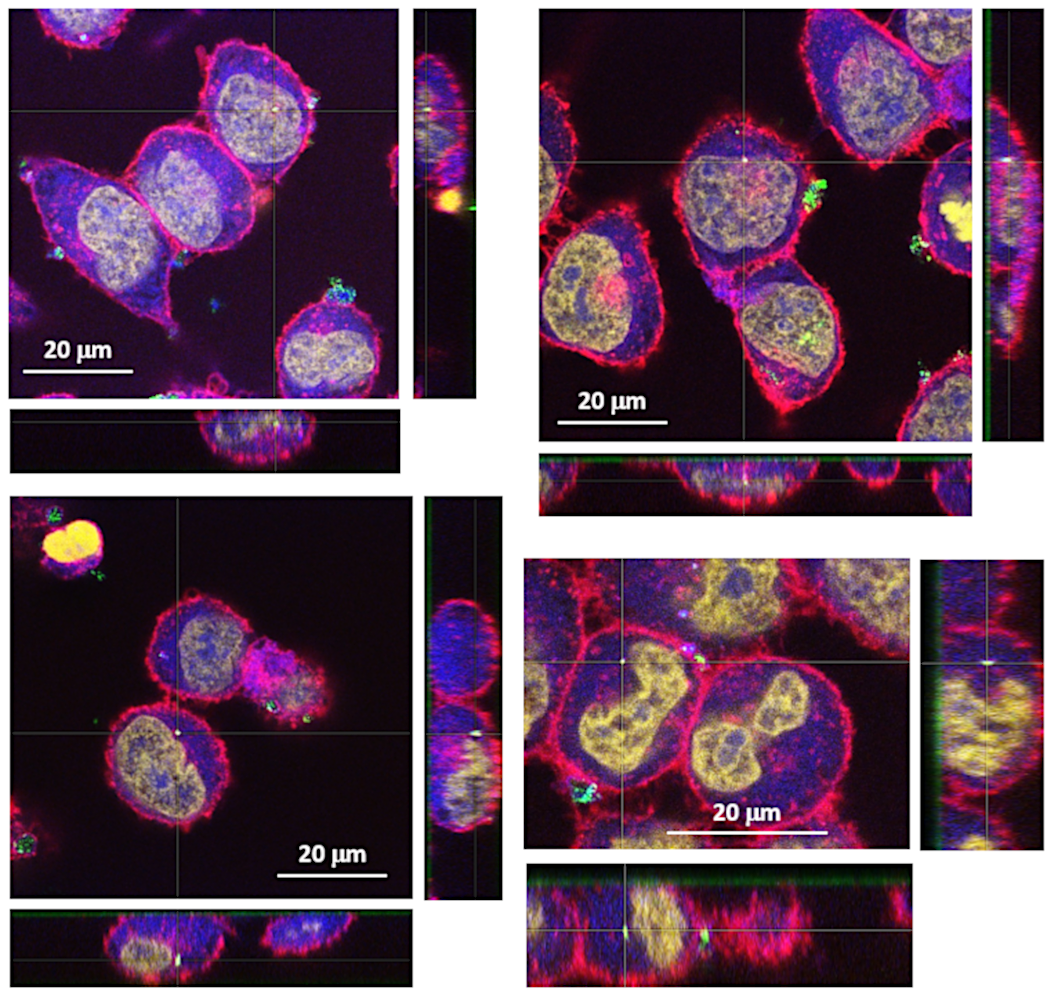

Supplement: Supplementary file 1 [file ijms-23-03410-s001.zip › ijms-1630965-supplementary.tif]
